# Postoperative Analgesic and Rehabilitation Outcomes After Implementation of a Kampo Plus Acetaminophen Pathway for Cervical Laminoplasty: A Retrospective Historical Control Study

**DOI:** 10.7759/cureus.117487

**Published:** 2026-10-05

**Authors:** Hiroyuki Motegi, Takaki Inoue, Shunsuke Suzuki

**Affiliations:** 1 Department of Orthopedic Surgery, Chiba Aoba Municipal Hospital, Chiba, JPN; 2 Department of Pharmacy, Chiba Aoba Municipal Hospital, Chiba, JPN

**Keywords:** acetaminophen, cervical laminoplasty, historical control, kampo medicine, nonsteroidal anti-inflammatory drugs, postoperative pain, rehabilitation

## Abstract

Background: Nonsteroidal anti-inflammatory drugs (NSAIDs) are widely used for acute postoperative pain but may be limited by adverse effects. In November 2023, our institution introduced a postoperative analgesic pathway using regular Kampo medicines plus acetaminophen, with NSAIDs administered as needed. This study compared postoperative pain, rescue NSAID use, and acute-phase rehabilitation outcomes after implementation of this pathway with those of the preceding scheduled-NSAID pathway.

Methods: This single-center retrospective historical-control study included 34 of 38 patients who underwent cervical laminoplasty at Chiba Aoba Municipal Hospital, Japan, between January 2022 and September 2025. The Kampo (K) group comprised 19 patients treated from November 2023 through September 2025 with Saireito (TJ-114), Keishibukuryogankayokuinin (TJ-125), and scheduled acetaminophen, with NSAIDs as needed. The historical NSAID (N) group comprised 15 patients treated from January 2022 through October 2023 with scheduled NSAIDs. Outcomes included Barthel Index (BI), time to first out-of-bed mobilization, postoperative length of stay, maximum postoperative and discharge Numerical Rating Scale (NRS) scores, maximum-to-discharge NRS change, and as-needed NSAID administrations. Exploratory regression analyses adjusted BI at discharge for age and baseline BI and adjusted NRS outcomes for age and preoperative neck pain Visual Analog Scale score.

Results: The K group was older than the N group (72 ± 11 vs. 65 ± 9 years; p = 0.033). Time to first out-of-bed mobilization and postoperative length of stay did not show statistically significant between-group differences. BI at discharge was lower in the K group in the unadjusted analysis (85 (75-90) vs. 100 (90-100); p = 0.030), whereas the adjusted difference (K minus N) was -2.78 points (95% CI, -19.22 to 13.66; p = 0.741). Maximum postoperative NRS (4 (3-5) vs. 3 (2.5-4.5); p = 0.159) and discharge NRS (0 (0-0.5) vs. 0 (0-2); p = 0.217) did not differ significantly. The exploratory maximum-to-discharge NRS change was larger in the K group (4 (3-4) vs. 2 (1.5-3); p = 0.004); the adjusted difference was 1.88 points (95% CI, 0.50-3.26; p = 0.008). As-needed NSAID administrations were 0 (0-1) and 0 (0-0), respectively (p = 0.259).

Conclusions: Implementation of the Kampo plus acetaminophen-based pathway was associated with a larger exploratory maximum-to-discharge NRS change, but not with lower as-needed NSAID use or clear differences in absolute maximum or discharge NRS scores. Because this was a small, noncontemporaneous historical-control study and the pathways differed in more than Kampo exposure, the findings do not establish an independent analgesic effect of Kampo medicines or equivalence in functional recovery. Prospective contemporaneous studies are warranted.

## Introduction

Nonsteroidal anti-inflammatory drugs (NSAIDs) are widely used for postoperative analgesia. However, their use may be limited by gastrointestinal, renal, and other adverse effects. Concerns regarding bone healing and fusion have also been reported in orthopedic and spinal fusion settings [1-4], although these findings are not directly applicable to nonfusion cervical laminoplasty. These considerations have encouraged interest in multimodal postoperative analgesic strategies that may reduce reliance on regularly administered NSAIDs.

Kampo medicines are traditional Japanese herbal formulations used for a variety of clinical conditions. Experimental and clinical studies have reported pharmacological effects related to fluid regulation, inflammation, and microcirculation for several Kampo formulations and their constituents [5-11]. Nevertheless, much of this evidence derives from nonpostoperative, animal, or laboratory settings, and clinical evidence supporting Kampo medicines specifically for acute pain after cervical laminoplasty remains limited. Accordingly, proposed mechanisms should not be assumed to translate directly into postoperative analgesic efficacy.

Following cervical laminoplasty, adequate pain control is important for early mobilization and acute-phase rehabilitation. In November 2023, our institution changed its postoperative analgesic pathway from regular NSAID administration to a regimen consisting of regular Saireito (TJ-114), Keishibukuryogankayokuinin (TJ-125), and acetaminophen, with NSAIDs administered as needed for pain [12]. Because acetaminophen was introduced together with the Kampo medicines, this clinical change represents a comparison between postoperative analgesic strategies rather than an isolated comparison of Kampo medicines with NSAIDs.

This retrospective historical-control study therefore compared patients treated before and after implementation of the Kampo plus acetaminophen-based pathway. We evaluated postoperative neck pain, as-needed NSAID use, and acute-phase rehabilitation outcomes, including Barthel Index (BI) scores, time to first out-of-bed mobilization, and postoperative length of stay. The objective was to examine associations between implementation of the revised analgesic pathway and these postoperative outcomes, rather than to establish an independent causal effect of Kampo medicines.

An abstract of this study was previously presented at the 62nd Annual Meeting of the Japanese Association of Rehabilitation Medicine in Kyoto, Japan, on June 14, 2025, based on 21 patients (11 in the Kampo medicine group and 10 in the pre-Kampo group). The present study includes additional patients identified during the study period.

## Materials and methods

Study design and setting

This single-center retrospective historical-control study included patients who underwent cervical laminoplasty at the Department of Orthopedic Surgery, Chiba Aoba Municipal Hospital, Chiba, Japan, between January 2022 and September 2025. Cervical laminoplasty was performed from C3 to C6 or from C3 to C7, and additional foraminotomy was performed in some patients. In November 2023, our institution changed its postoperative analgesic pathway from regular NSAID administration to a regimen consisting of regular Kampo medicines and acetaminophen, with NSAIDs administered as needed for pain. We did not identify any clear changes during the study period in surgical technique, postoperative rehabilitation or mobilization protocols, nursing care, or other perioperative management apart from the change in the postoperative analgesic pathway.

​Inclusion and exclusion criteria, and sample size

Of the 38 patients identified during the study period, 34 patients (23 men and 11 women) were included in the analysis. One patient who received no oral analgesic medication and three patients who regularly received both NSAIDs and Kampo medicines were excluded. The 19 patients treated from November 2023 through September 2025 after implementation of the revised postoperative analgesic pathway were assigned to the Kampo plus acetaminophen group (K group). The 15 patients treated from January 2022 through October 2023 before implementation of this pathway constituted the historical NSAID group (N group) (Figure 1).

**Figure 1:**
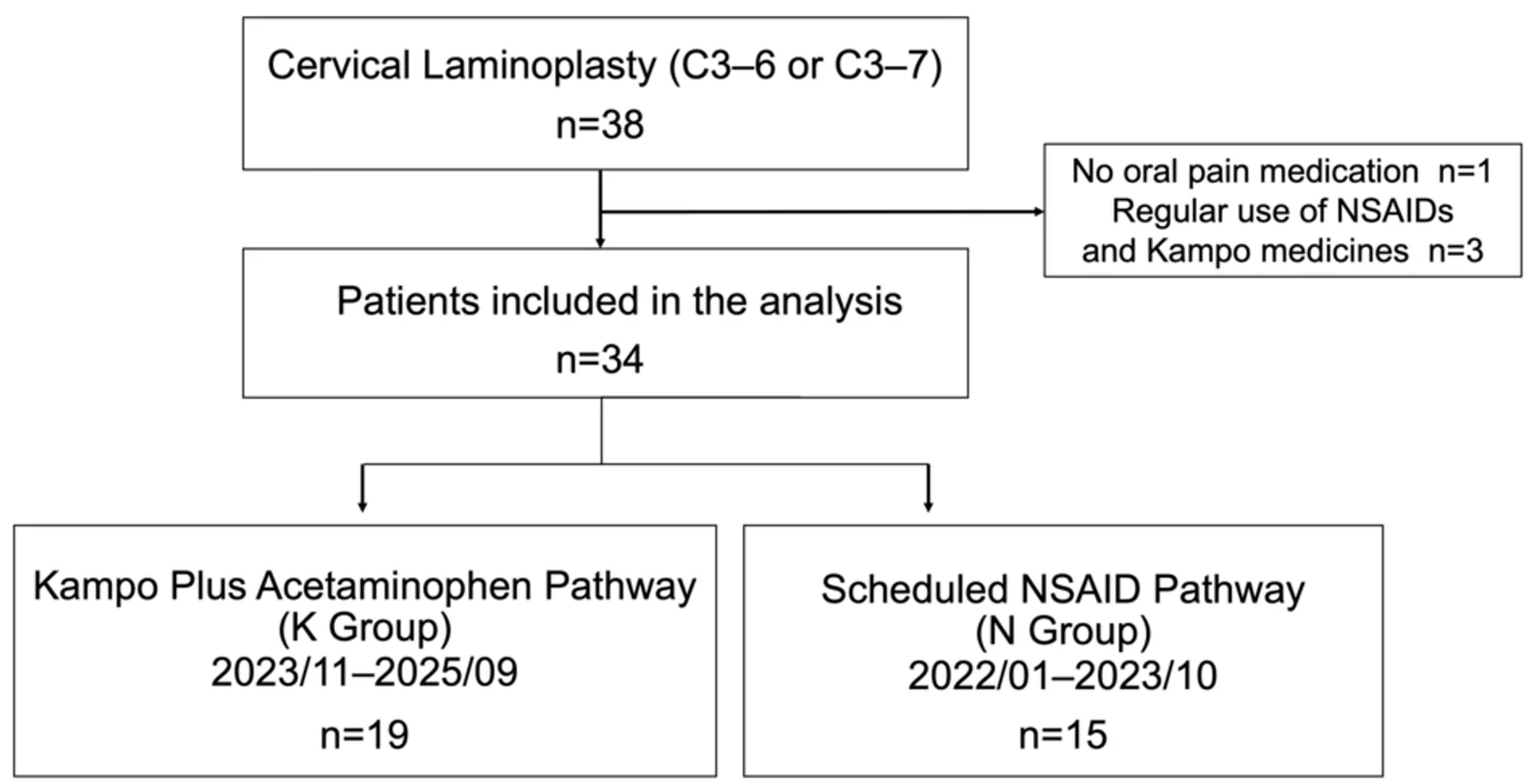
Study design and patient selection Flowchart of patient selection and allocation to the Kampo medicine group (K group) and NSAID group (N group). Of the 38 patients initially identified, four were excluded, leaving 34 patients for the final analysis NSAIDs: nonsteroidal anti-inflammatory drugs

In the K group, beginning on the day after surgery, patients regularly received two packets each of Saireito (TJ-114) and Keishibukuryogankayokuinin (TJ-125) twice daily after breakfast and dinner, together with acetaminophen 500 mg three times daily after meals [12]. For breakthrough pain, lornoxicam 4 mg with rebamipide 100 mg or a diclofenac sodium suppository 50 mg was administered as needed.

In the N group, beginning on the day after surgery, patients regularly received lornoxicam 4 mg and rebamipide 100 mg three times daily after meals. A diclofenac sodium suppository 50 mg was administered as needed for pain.

Data collection

The study variables included age, sex, diagnosis, blood loss, operative time, preoperative Japanese Orthopedic Association (JOA) score [13], level of laminoplasty, presence of additional foraminotomy, and preoperative neck pain Visual Analog Scale (VAS) score. Acute-phase rehabilitation outcomes included time to first out-of-bed mobilization after surgery, postoperative length of stay, and BI [14] scores at the beginning of acute-phase rehabilitation and at discharge. The BI improvement rate was calculated as follows: \(\frac{\left(\mathrm{BI}_{\text{discharge}}-\mathrm{BI}_{\text{beginning of acute-phase rehabilitation}}\right)} {100-\mathrm{BI}_{\text{beginning of acute-phase rehabilitation}}} \times 100\). One patient in the N group had a BI score of 100 at both the beginning of acute-phase rehabilitation and discharge, resulting in a denominator of zero; therefore, this patient was excluded only from the BI improvement-rate analysis.

Postoperative neck pain was assessed using the Numerical Rating Scale (NRS) [15]. We evaluated the maximum postoperative NRS score recorded from the day after surgery through discharge, the NRS score at discharge, and the maximum-to-discharge NRS change, calculated as the maximum postoperative NRS score minus the NRS score at discharge. The maximum-to-discharge NRS change was treated as an exploratory derived outcome. Because NRS measurements were obtained during routine clinical care rather than at standardized postoperative time points, fixed-time longitudinal NRS analyses were not performed. The number of as-needed NSAID administrations from the day after surgery through discharge was determined from the medical and medication administration records and represented actual administrations rather than prescriptions.

​Statistical analysis

Age was summarized as mean ± standard deviation and compared between groups using an unpaired t-test. Sex was compared using Fisher's exact test. Blood loss, operative time, preoperative JOA score, preoperative neck pain VAS score, time to first out-of-bed mobilization, postoperative length of stay, BI scores, BI improvement rate, maximum postoperative NRS score, NRS score at discharge, maximum-to-discharge NRS change, and the number of as-needed NSAID administrations were summarized as median (Q1-Q3), as appropriate, and compared between groups using the two-sided Mann-Whitney U test. Standardized mean differences (SMDs) were calculated to describe between-group differences in baseline and perioperative characteristics.

Because this was a retrospective exploratory study, no primary outcome was prospectively prespecified, no a priori sample-size calculation was performed, and no adjustment for multiple comparisons was applied. All eligible patients during the predefined study period were included, and all evaluated outcomes were interpreted as exploratory. Exploratory multivariable linear regression analyses with HC3 robust standard errors were performed to examine the association between group and selected outcomes while accounting for major measured baseline differences. BI at discharge was adjusted for age and BI at the beginning of acute-phase rehabilitation, whereas NRS outcomes were adjusted for age and preoperative neck pain VAS score. Adjusted group differences are reported with 95% confidence intervals (CIs).

Initial statistical analyses were performed using ystat2008 (version 2008; developed by Shinya Yamazaki, Tokyo, Japan) [16]. Additional analyses, including SMDs and exploratory multivariable regression analyses, were performed using Python (Python Software Foundation, Wilmington, DE) with SciPy (version 1.17.0; SciPy Developers; NumFOCUS, Austin, TX) and statsmodels (version 0.14.6; statsmodels Developers; NumFOCUS, Austin, TX). All statistical tests were two-sided, and p < 0.05 was considered statistically significant.

## Results

Patient characteristics and perioperative variables

A total of 34 patients were included in the analysis, comprising 19 patients in the K group and 15 in the N group. The mean age was significantly higher in the K group than in the N group (72 ± 11 vs. 65 ± 9 years, p = 0.033). The proportions of male patients were 63% and 73%, respectively (p = 0.715). SMDs were also calculated to describe between-group differences in baseline and perioperative characteristics. The SMD for age was 0.768, whereas the SMDs for sex and preoperative JOA score were 0.220 and 0.013, respectively. The SMDs for blood loss and operative time were 0.612 and 0.170, respectively. Additional baseline and perioperative characteristics, including diagnosis, preoperative neck pain VAS score, laminoplasty level, and additional foraminotomy, are presented in Table 1.

**Table 1:** Baseline characteristics and perioperative variables of the K and N groups Data are presented as mean ± SD, median (Q1-Q3), or n (%), as appropriate. Age was compared using an unpaired t-test, sex using Fisher’s exact test, and continuous variables with nonnormal distributions using the Mann-Whitney U test. SMDs are presented to describe between-group differences in baseline and perioperative characteristics. A dash (-) indicates that a statistical test was not performed or was not applicable VAS: Visual Analog Scale; JOA: Japanese Orthopaedic Association; SD: standard deviation; SMD: standardized mean difference

Variable	K group (n = 19)	N group (n = 15)	Test statistic	p value	SMD
Age, years, mean ± SD	72 ± 11	65 ± 9	t = 2.22	0.033	0.768
Male sex, n (%)	12 (63)	11 (73)	-	0.715	0.220
Diagnosis, n (%)					
Cervical spondylotic myelopathy	14 (73.7)	9 (60.0)	-	-	0.294
Cervical radiculopathy	2 (10.5)	3 (20.0)	-	-	0.266
Ossification of the posterior longitudinal ligament	3 (15.8)	3 (20.0)	-	-	0.110
Preoperative neck pain VAS, median (Q1-Q3)	2.7 (0.5-6.75)	4.3 (2.15-5.0)	U = 128.5	0.639	0.013
Blood loss (mL), median (Q1-Q3)	42 (10-50)	58 (1-146)	U = 109.5	0.252	0.612
Operative time (minutes), median (Q1-Q3)	201 (164.5-222)	190 (156.5-215)	U = 136.0	0.835	0.170
Preoperative JOA score, median (Q1-Q3)	9.5 (7.5-12.25)	9 (8-12.25)	U = 141.0	0.972	0.013
C3-7 laminoplasty, n (%)	13 (68.4)	7 (46.7)	-	-	0.451
Additional foraminotomy, n (%)	6 (31.6)	4 (26.7)	-	-	0.108

BI and acute-phase rehabilitation

Time to first out-of-bed mobilization and postoperative length of stay did not differ significantly between groups. The BI score at discharge was lower in the K group than in the N group (median (Q1-Q3), 85 (75-90) vs. 100 (90-100), p = 0.030). After recalculation using the formula stated in the Methods section, the BI improvement rate was 71.4% (47.2-85.4) in the K group and 94.4% (80.8-100.0) in the N group (p = 0.057). One patient in the N group was excluded only from the BI improvement-rate analysis because the denominator of the formula was zero. In an exploratory multivariable analysis adjusted for age and BI at the beginning of acute-phase rehabilitation, the adjusted group difference in BI at discharge (K minus N) was -2.78 points (95% CI, -19.22 to 13.66; p = 0.741) (Table 2).

**Table 2:** BI and acute-phase rehabilitation outcomes Data are presented as median (Q1-Q3). Between-group comparisons were performed using the two-sided Mann-Whitney U test ^*^p < 0.05 ^†^The BI improvement rate was calculated as ((BI at discharge - BI at the beginning of acute-phase rehabilitation) / (100 - BI at the beginning of acute-phase rehabilitation)) × 100. One patient in the N group had a BI score of 100 at both the beginning of acute-phase rehabilitation and discharge, resulting in a denominator of zero; therefore, this patient was excluded only from the BI improvement-rate analysis (K group, n = 19; N group, n = 14) BI: Barthel Index

Outcome	K group (n = 19)	N group (n = 15)	Test statistic	p value
Time to first out-of-bed mobilization, days	2 (2-2.5)	2 (2-2)	U = 120.0	0.382
Postoperative length of stay, days	16 (13-19.5)	13 (11-17.5)	U = 120.0	0.443
BI at the beginning of acute-phase rehabilitation	20 (10-60)	15 (10-65)	U = 130.0	0.674
BI at discharge	85 (75-90)	100 (90-100)	U = 81.0	0.030^*^
BI improvement rate^†^, %	71.4 (47.2-85.4)	94.4 (80.8-100.0)	U = 81.0	0.057

Postoperative NRS scores and NSAID use

The maximum postoperative NRS score and NRS score at discharge did not differ significantly between groups (p = 0.159 and p = 0.217, respectively). The maximum-to-discharge NRS change was greater in the K group than in the N group (median (Q1-Q3), 4 (3-4) vs. 2 (1.5-3), p = 0.004). In exploratory multivariable analyses adjusted for age and preoperative neck pain VAS score, the adjusted group differences (K minus N) were +1.02 points (95% CI, -0.29 to 2.34; p = 0.128) for maximum postoperative NRS, -0.86 points (95% CI, -2.04 to 0.32; p = 0.152) for NRS at discharge, and +1.88 points (95% CI, 0.50-3.26; p = 0.008) for maximum-to-discharge NRS change. The number of as-needed NSAID administrations did not differ significantly between the groups (median (Q1-Q3), 0 (0-1) vs. 0 (0-0), p = 0.259) (Table 3).

**Table 3:** Postoperative neck pain and as-needed NSAID use Data are presented as median (Q1-Q3). Between-group comparisons were performed using the two-sided Mann-Whitney U test ^*^p < 0.05 ^†^Maximum-to-discharge NRS change was calculated as the maximum postoperative NRS score minus the NRS score at discharge and was treated as an exploratory derived outcome NRS: Numerical Rating Scale; NSAID: nonsteroidal anti-inflammatory drug; PRN: as needed

Outcome	K group (n = 19)	N group (n = 15)	Test statistic	p value
Maximum postoperative NRS	4 (3-5)	3 (2.5-4.5)	U = 102.5	0.159
NRS at discharge	0 (0-0.5)	0 (0-2)	U = 112.5	0.217
Maximum-to-discharge NRS change^†^	4 (3-4)	2 (1.5-3)	U = 61.5	0.004^*^
PRN NSAID administrations	0 (0-1)	0 (0-0)	U = 115.0	0.259

## Discussion

This retrospective historical-control study compared two postoperative analgesic strategies after cervical laminoplasty: a Kampo plus acetaminophen-based pathway with NSAIDs administered as needed and the preceding pathway based on regular NSAID administration. In the unadjusted analysis, BI at discharge was lower in the K group than in the N group, whereas time to first out-of-bed mobilization, postoperative length of stay, and BI improvement rate did not show statistically significant between-group differences. In the exploratory analysis adjusted for age and BI at the beginning of acute-phase rehabilitation, the between-group difference in BI at discharge was attenuated and imprecise (K minus N, -2.78 points; 95% CI, -19.22 to 13.66; p = 0.741). These findings should not be interpreted as demonstrating equivalence in functional recovery because of the small sample size and wide CI.

For postoperative neck pain, neither the maximum postoperative NRS score nor the NRS score at discharge differed significantly between groups in the unadjusted analyses. The maximum-to-discharge NRS change was larger in the K group, and this association persisted in an exploratory regression model adjusted for age and preoperative neck pain VAS (adjusted difference, 1.88 points; 95% CI, 0.50-3.26; p = 0.008). In contrast, the adjusted between-group differences in maximum postoperative NRS and NRS at discharge were not statistically significant. Because the maximum-to-discharge change was a derived outcome based on the highest NRS recorded during hospitalization rather than a fixed postoperative time point, it may be influenced by length of stay and measurement frequency. Accordingly, this finding should be considered exploratory and does not establish an independent analgesic effect of Kampo medicines.

The number of as-needed NSAID administrations did not differ significantly between the groups. However, this measure does not represent total NSAID exposure: patients in the N group received scheduled lornoxicam, whereas patients in the K group received scheduled acetaminophen and used NSAIDs as rescue medication. The type and potency of rescue NSAIDs also differed, and the observation period depended on postoperative length of stay. Therefore, the present data should be interpreted as a comparison of postoperative analgesic strategies rather than as evidence that Kampo medicines reduced overall NSAID exposure.

A previous case report described integrative Korean medicine, including herbal treatment and acupuncture, in a patient with severe cervical spondylotic myelopathy [17]; however, such evidence is not directly comparable with acute postoperative pain management after cervical laminoplasty. To our knowledge, clinical evidence specifically evaluating Kampo-based postoperative analgesic pathways after cervical laminoplasty remains limited. The present findings therefore provide exploratory clinical observations rather than confirmatory evidence of efficacy.

Saireito (TJ-114) has been investigated for effects related to fluid regulation and inflammation [7,8,10,18], whereas Keishibukuryogan and its constituents have been studied in relation to microcirculation, inflammation, and other pharmacological effects [5,6,9,11,19]. Some of the supporting evidence derives from animal, cellular, or other nonpostoperative models and therefore cannot establish the mechanism of any clinical effect observed in this cohort. Moreover, this study did not directly measure postoperative edema, hematoma formation, inflammatory biomarkers, or microcirculatory changes. Any proposed mechanistic relationship between these pharmacological findings and postoperative neck pain after cervical laminoplasty should therefore be regarded as hypothetical.

Several limitations should be considered. First, this was a single-center retrospective historical-control study with a small sample size, and treatment allocation was determined by calendar period rather than contemporaneous randomization. Secular changes in perioperative care, rehabilitation, clinical practice, or other unmeasured factors may therefore have contributed to the observed differences. Although no clear changes in surgical technique, postoperative rehabilitation or mobilization protocols, nursing care, or perioperative management other than the postoperative analgesic pathway were identified from the available records and our knowledge of institutional practice, undocumented or unrecognized secular changes cannot be excluded. Second, the K group was older, and other baseline or perioperative differences were present, as reflected by the SMDs. Exploratory multivariable analyses were performed with a deliberately limited number of covariates because of the small sample size, but residual and unmeasured confounding cannot be excluded. Variables such as comorbidities, renal or gastrointestinal risk, preoperative analgesic exposure, perioperative anesthesia and analgesia, and rehabilitation exposure were not comprehensively evaluated in the present dataset. Third, the study included patients with different cervical diagnoses, and preoperative JOA scores may not fully capture differences in pain or neurologic manifestations among these conditions. Fourth, the postoperative analgesic regimens differed in more than Kampo exposure because the K group also received scheduled acetaminophen, whereas the historical N group received scheduled NSAIDs. The study therefore cannot isolate an independent effect of Kampo medicines. Fifth, the maximum-to-discharge NRS change was an exploratory derived measure that may be affected by hospitalization duration and the frequency and timing of NRS assessments. Finally, the number of as-needed NSAID administrations did not account for total NSAID dose, drug potency, or exposure time. No a priori sample-size calculation was performed, no primary outcome was prospectively prespecified, and multiple outcomes were evaluated without adjustment for multiple comparisons; therefore, chance findings cannot be excluded. These limitations restrict causal inference and the generalizability of the findings.

## Conclusions

This retrospective historical-control study found that implementation of a Kampo plus acetaminophen-based postoperative analgesic pathway was associated with a larger maximum-to-discharge NRS change than the preceding regular NSAID-based pathway, whereas maximum postoperative NRS, NRS at discharge, and as-needed NSAID administrations did not differ significantly between groups. Functional recovery outcomes did not demonstrate equivalence between the two strategies, and the adjusted difference in BI at discharge was imprecise. Because the treatment regimens differed in both Kampo and acetaminophen exposure and were implemented during different calendar periods, these findings should be interpreted as exploratory associations and do not establish an independent analgesic effect of Kampo medicines. Larger prospective studies are warranted to evaluate these postoperative analgesic strategies.
